# Editorial: Nutrition of the premature neonate: Physiology, pathology and management of the immature and developing gut

**DOI:** 10.3389/fped.2022.1039153

**Published:** 2022-11-11

**Authors:** Letizia Capasso, Francesco Raimondi

**Affiliations:** Neonatology, Department of Translational Medical Sciences, Università Federico II di Napoli, Naples, Italy

**Keywords:** nutrition, preterm, feeding (in)tolerance, developing gut, gut microbioma, neonate, premature

**Editorial on the Research Topic** Nutrition of the premature neonate: Physiology, pathology and management of the immature and developing gut By Capasso L and Raimondi F. (2022) Front. Pediatr. 10: 1039153. doi: 10.3389/fped.2022.1039153

The nutritional requirements of preterm and very preterm neonates are a fundamental element of their management. Optimal nutrition improves growth, neurodevelopmental progress, and may play a role in the development of prematurity-related pathologies such as infection/sepsis, retinopathy of prematurity, and Bronchopulmonary dysplasia.

However, despite greater knowledge in the field, the development of new devices and solutions for parenteral nutrition, modern preterm formulas, and molecules to ameliorate immunomodulation of the immature gut, optimizing the nutrition of preterm neonates remains a challenge.

The pathogenesis of malnutrition in preterm infants is multifactorial, depending on both their wellbeing *in utero* and their clinical course in NICU. This Research Topic aims to expand the knowledge on this issue.

It has been postulated that antenatal antibiotics (AAB) affect the colonization of both the fetal and neonatal guts. This compromises the gut microbiome in preterm infants and may impair both gut development and NEC. Such an unfavourable microbiome affects the progress of enteral feeding and, consequently, body growth. Luo et al. investigated the roles of prenatal antibiotic exposure and neonatal enteral feeding. The results showed that body growth varied according to the exposure levels of AAB and GA in infants.

A crucial phase of body growth is the transition from parenteral to enteral nutrition during clinical course in NICU. Wang et al. reviewed nutrition practices in extremely preterm infants, with a focus on the transition from parenteral to enteral feeding and their association with growth. The authors confirmed that the total protein intake (parenteral and enteral) was a primary factor affecting postnatal growth failure. Lin et al. demonstrated that the addition of human milk fortifiers to a specific feed volume may be the best practice for promoting growth and preventing extrauterine growth restriction (EUGR) in very preterm neonates. The incidence of EUGR and related conditions in very preterm infants is discussed more extensively by Shen et al.

Alexander et al. also investigated the transition from parenteral to enteral nutrition in late preterm infants.

Nutritional support from birth may influence microbiome acquisition. Tadros et al. investigated how postnatal gut microbial colonization, which is modifiable, is associated with childhood growth in preterm infants. Young et al. discussed a method to characterize the composition of bacteriophage in breast milk in order to determine its clinical relevance for the development of a healthy gut microbiome in very preterm infants. These studies are based on the assumption that a healthy gut microbiome influences healthy growth.

